# Are Known Cyanotoxins Involved in the Toxicity of Picoplanktonic and Filamentous North Atlantic Marine Cyanobacteria?

**DOI:** 10.3390/md8061908

**Published:** 2010-06-21

**Authors:** Bárbara Frazão, Rosário Martins, Vitor Vasconcelos

**Affiliations:** 1 Departamento de Biologia, Faculdade de Ciências da Universidade do Porto, 4169-007 Porto, Portugal; E-Mail: bmfrazao@gmail.com (B.F.); 2 Centro Interdisciplinar de Investigação Marinha e Ambiental, CIIMAR/CIMAR, 4050-123 Porto, Portugal; E-Mail: mrm@estsp.ipp.pt (R.M.); 3 IBMC-Instituto de Biologia Molecular e Celular, Universidade do Porto, 4150-180 Porto, Portugal; 4 Escola Superior de Tecnologia da Saúde do Porto, Instituto Politécnico do Porto, 4400-330 Vila Nova de Gaia, Portugal

**Keywords:** marine cyanobacteria, cyanotoxins, ecotoxicology, Artemia salina, toxin genes

## Abstract

Eight marine cyanobacteria strains of the genera *Cyanobium*, *Leptolyngbya*, *Oscillatoria*, *Phormidium*, and *Synechococcus* were isolated from rocky beaches along the Atlantic Portuguese central coast and tested for ecotoxicity. Strains were identified by morphological characteristics and by the amplification and sequentiation of the 16S rDNA. Bioactivity of dichloromethane, methanol and aqueous extracts was assessed by the *Artemia salina* bioassay. Peptide toxin production was screened by matrix assisted laser desorption/ionization time of flight mass spectrometry. Molecular analysis of the genes involved in the production of known cyanotoxins such as microcystins, nodularins and cylindrospermopsin was also performed. Strains were toxic to the brine shrimp *A. salina* nauplii with aqueous extracts being more toxic than the organic ones. Although mass spectrometry analysis did not reveal the production of microcystins or other known toxic peptides, a positive result for the presence of *mcyE* gene was found in one *Leptolyngbya* strain and one *Oscillatoria* strain. The extensive brine shrimp mortality points to the involvement of other unknown toxins, and the presence of a fragment of genes involved in the cyanotoxin production highlight the potential risk of cyanobacteria occurrence on the Atlantic coast.

## 1. Introduction

Cyanobacteria are known for their diversity in terms of morphological, physiological and toxicological properties. Lately, the production of bioactive compounds with commercial and medical applications has also increased interest in these organisms [1]. In fact, together with the production of potent toxins, cyanobacteria produce many substances interesting in terms of antifungal, antibiotic and anticancerigenous activities [2,3].

The occurrence and ecotoxicology of cyanobacteria are particularly well documented in freshwater habitats [4,5]. In contrast, studies related to marine environments have not been performed to the same extent and are mainly from tropical regions, where cyanobacteria mass occurrences have been reported [6,7]. As described for cyanobacteria blooms in freshwater and brackish waters, the increase in cyanobacteria bloom formation registered in coastal areas has been attributed to factors such as high irradiation, high temperatures and increased nutrient loading, as a consequence of human population growth near these locations [8–11]. Similar to other littoral areas of the world, the Portuguese coast has been subjected to an increased human pressure and, consequently, coastal eutrophication. Eutrophication, powered by the phenomenon of global warming, increases the potential of marine cyanobacteria proliferation. As most coastal areas have a considerable importance for biodiversity, fish industry and tourism, it is of significant importance to also study the putative toxicity of cyanobacteria that occurs in this marine environment. Moreover, previous studies conducted with marine cyanobacteria isolated from north Portuguese rocky beaches, reported that strains of the genera *Aphanotece*, *Oscillatoria*, *Phormidium*, *Synechococcus* and *Synechocystis* caused toxic effects on mammals, marine invertebrates, growth inhibition of Gram-positive bacteria and cytotoxicity in human cell lines [12–15].

In this work we evaluated the potential toxicity of marine cyanobacteria isolated from the central coast of Portugal by means of biological, chemical and molecular biology analysis.

## 2. Materials and Methods

### 2.1. Cyanobacteria strains

Marine cyanobacteria strains were isolated from water samples and solid materials collected from rocky beaches on the central coastal area of Portugal [12] (Table 1). Strains were isolated and maintained in Z8 medium [16] supplemented with 20 g NaCl/L. Unicyanobacterial cultures were performed in 4 L Z8 medium at 25 ºC, irradiation of 10 μmol photons s^−1^ m^−2^ was provided by cool white fluorescent tubes and with a light/dark cycle of 14 h/10 h. Cells were harvested after one-month of growth by decantation for the non-floating strains, centrifugation for the floating strains and filtration through a 3 μm pore net for filamentous strains. Cyanobacterial biomass was washed repeatedly with deionized water to remove the excess of NaCl. Samples were frozen and freeze-dried and the material was stored at −20 ºC.

### 2.2. Identification of cyanobacteria isolates

Cyanobacteria isolates were identified by their morphology and size of cells and colonies and by a molecular approach. Phenotypic identification was based on the phycological and bacterial guides [17–21]. Molecular identification of the isolates was based on PCR amplification and sequencing of the 16S rDNA gene.

#### Molecular identification

Total genomic DNA was extracted from freeze dried biomass using the commercial DNA extraction kit AquaPure kit (Bio-Rad, Hercules, USA) and following the manufacturer’s instructions. The set of primers 27F/809R and 740F/1494R were used for PCR amplification of the cyanobacteria 16S rDNA gene [22,23] (Table 2). Thermal cycling program was based on the work by Jungblut *et al.* [23], with the exceptions that 35 cycles were preformed with a final phase extension of 72 ºC for 5 min. Reactions were carried out in a 100 μL reaction volume that consisted of 1X Tampon solution (Bioline, 10x NH_4_ Reaction Buffer), 2.5 μL dNTPs (Bioline, 2.5 mM), 2.5 mM MgCl_2_ (Bioline, 50 mM), 0.5 pmol of each primer (10 pM/μL), 2.5 U of Taq DNA polimerase (Bioline), 69.5 μl sterile ultra-pure water and 2.5 μL of DNA sample. PCR products were extracted from gel with the Jet Quick Spin Column Technique kit (Genomed) according to the manufacturer’s instructions and sent for direct sequencing to STABVIDA®. Automated sequencing was performed using the BigDye Terminator Kit by Applied Biosystems and the 96-capillary 3730xL DNA Analyzer by Applied Biosystems. Sequences were analyzed using the BLAST system (http://www.ncbi.nlm.nih.gov/BLAST/).

### 2.3. Toxin genes detection

In order to determine the presence of genes implicated in the production of cyanotoxins, we applied a range of molecular primers that are currently used for detection of genes involved in the production of microcystins (MC), nodularins (NOD) and cylindrospermopsin (CYN) (Table 2). The MC gene cluster, *mcy*, comprises 10 genes in two transcribed operons, *mcyA-C* and *mcyD-J* [24]. The NOD gene cluster, *nda*, consists of nine open reading frames (*ndaA-I*) [25]. For the detection of genes involved in MC and NOD production we used the HEP primers set. This primers have as target sequences the aminotransferase (AMT) domain, which is located on the modules *mcyE* and *nda*F of the MC and NOD synthetase enzyme complexes, respectively [24,26]. For the detection of other genes implied in the production of MC, we used the primers *mcyA-C*, which detect the *mcyA*, *mcyB* and *mcyC* genes [27,28]. For detection of genes involved in the CYN production we used the polyketide synthase PKS M4 and M5 primers and the peptide synthetase M13 and M14 primers designed by Schembri *et al.* [29] who demonstrate a direct link between the presence of the polyketide synthase and peptide synthetase genes and the ability of cyanobacteria to produce CYN.

The conditions of the PCR reactions were as those described for the amplification of the 16S rRNA gene. Concerning the cycling conditions, for *mcyA*-Cd genes the thermal cycling conditions were 1 cycle at 95 ºC for 2 min, 35 cycles at 95 ºC for 90 s, 56 ºC for 30 s and 72 ºC for 50 s and 1 cycle at 72 ºC for 7 min. For *HEP* genes, the thermal cycling conditions were 1 cycle at 95 ºC for 2 min, 35 cycles at 95 ºC for 90 s, 55 ºC for 30 s and 72 ºC for 1 min and 1 cycle at 72 ºC for 7 min. For CYL genes, the thermal cycling conditions were 1 cycle at 95 ºC for 2 min, 35 cycles at 95 ºC for 90 s, 55 ºC for 30 s and 72 ºC for 1 min and 1 cycle 72 ºC for 7 min [27,28]. PCR products were purified as described previously for the 16S rRNA gene amplification. As a positive control for MC and NOD, a *Microcystis aeruginosa* strain from Lagoa de Mira, Portugal (M6 strain), and for CYN a *Cylindrospermopsis raciborskii* strain from Queensland, Australia (AQS strain) were used.

### 2.4. Peptides screening by MALDI-TOF MS

The production of oligopeptides such as MC was screened by matrix assisted laser desorption/ionization time of flight mass spectrometry (MALDI-TOF MS). Freeze dried biomass from the marine strains was screened for the production of oligopeptides by MALDI-TOF MS as described in Welker *et al.* [30]. By this analysis, several hundreds of different oligopeptides of different types can potentially be detected and identified by post-source-decay fragmentation.

### 2.5. Cyanobacteria extracts for *Artemia salina* bioassay

Cyanobacteria freeze-dried biomass (100 mg/mL) was extracted with a diclorometane:methanol (50:50; v:v) solution (dichloromethane extract), a 80% methanol solution (methanolic extract) and deionised water (aqueous extract). After solvent addition, solutions were sonicated for 3 × 20 s on ice and kept in the dark for 12 h for the dichloromethane extract and for 1.5 h for the methanolic and aqueous extracts. Extracts were centrifuged at 23 000 × *g* for 10 min. Supernatants were collected, evaporated to dryness and ressuspended in filtered natural seawater. Extracts were diluted with filtered natural seawater (75, 50 and 25%) and then filtered through a 0.22 μm filter.

### 2.6. *Artemia salina* bioassay

*A. salina* bioassay was performed in 96 wells polystyrene plates [31]. *Artemia* cysts (available in aquaristic shops) were incubated in 1 L of natural filtered seawater for 24 h. Cysts were maintained at 25 ºC with continuous light and aeration. Ten to fifteen individuals were placed into 200 μL of test solution in triplicate. Control was performed with natural filtered seawater. Plates were incubated in the dark for 48 h, with very low agitation. After 24 and 48 h, dead nauplii were counted and by the end of the assay total numbers of individuals were registered. Results were expressed as percentage of mortality and LC_50_ was calculated using the statistic software 8.0 of StatSoft Inc. 1984–2007 version.

## 3. Results

### 3.1. Cyanobacteria strains

Eight cyanobacteria strains were successfully isolated and studied (Table 1). Strains were isolated from seawater and solid material obtained from rock scrapings and *Patella* sp. shells. Taxonomic identification, based on morphology and on the sequencing of the 16S rRNA gene followed by a BLAST search, led to the coccoid *Cyanobium* and *Synechococcus* and to the filamentous *Leptolyngbya*, *Oscillatoria* and *Phormidium*. Strains code and the accession numbers of 16S rDNA sequences are presented in Table 1.

### 3.2. Detection of genes involved in toxin production

**Figure 1:**
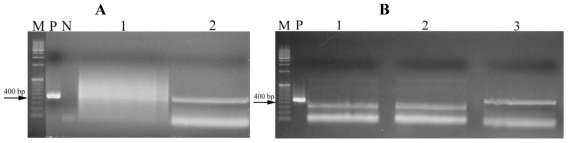
PCR products amplified from strains LEGE 06010 and LEGE 06018, *Leptolyngbya* sp. and *Oscillatoria* sp, respectively, with HEPF/HEPR primers. (**A**) Lane 1: strain LEGE 06025 (data not shown); Lane 2: strain LEGE 06010; M-1Kb; (**B**) Lanes 1 and 2: strain LEGE 06025 (data not shown); Lane 3: strain LEGE 06018. M: Marker; P: Positive control (M6 strain), N: Negative control.

For most of the strains, no amplification of any of the toxin genes was obtained. Nevertheless, 439 and 431 base pair (bp) fragments were obtained with the HEP primers reaction for strains LEGE 06010 and LEGE 06018, *Leptolyngbya* sp. and *Oscillatoria* sp. respectively (Figure 1). In order to confirm the identity of the amplified fragments, the PCR products were sequenced. The sequences were compared with sequences from the GenBank and we found 99% similarity with the *mcyE* of the microcystin synthetase gene cluster from a *Microcystis* sp. CYN06 strain (Table 3).

### 3.3. MALDI-TOF MS

Mass signals of known or putative peptides such as MC were not found for any of the eight tested strains. In all mass spectra, peaks at *m/z* 871 and *m/z* 593 for pheophytin and pheophorbide, respectively, were present, indicating that the extraction of the cells was efficient.

### 3.4. *Artemia salina* assay

**Figure 2:**
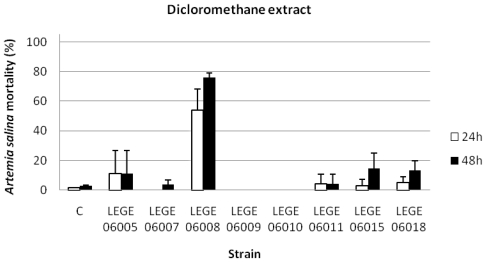
Mortality of *Artemia salina* (%) after 24 and 48 h exposure to dichloromethane extracts at a concentration of 100 mg/mL (C: control).

**Figure 3:**
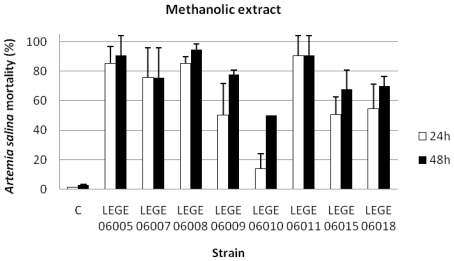
Mortality of *Artemia salina* (%)after 24 and 48 h exposure to the methanolic extracts at a concentration of 100 mg/mL (C: control).

**Figure 4:**
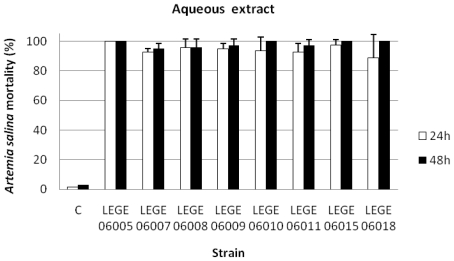
Mortality of *Artemia salina* (%) after 24 and 48 h exposure to the aqueous extracts at a concentration of 100 mg/mL (C: control).

The results concerning the percentage of *A. salina* mortality exposed to the dichloromethane (Figure 2), the methanolic (Figure 3) and the aqueous extracts (Figure 4), showed that for the dichloromethane extract only the strain LEGE 06008 revealed mortality higher than 50%. Results concerning the methanolic and the aqueous extract were more pronounced for most of the strains, with strains LEGE 06005, LEGE 06010, LEGE 06015 and LEGE 06018 reaching 100% mortality with the aqueous extract. The average 24 h and 48 h LC_50_ for the methanolic extract were 50.8 mg/mL and 50.2 mg/mL, and the 24 h and 48 h LC_50_ for aqueous extract were on average 50.7 mg/mL and 50.5 mg/mL, respectively (Table 4).

## 4. Discussion

Marine cyanobacteria blooms are mostly reported from tropical and subtropical regions in spite that cyanobacteria blooms in freshwater ecosystems are common worldwide. In the absence of large amounts of biomass from natural samples, the isolation of strains and cultivation under laboratory conditions remains essential to study the putative toxicity of strains and to infer about the potential threat to humans and to ecosystems. In this work, we found that aqueous and organic extracts of marine cyanobacteria from the coccoid genera *Cyanobium* and *Synechococcus* and the filamentous genera *Leptolyngbya*, *Oscillatoria* and *Phormidium* isolated from the Portuguese coast induced acute toxicity in nauplii of the brine shrimp *A. salina*. Toxicity revealed by the nauplii mortality was evident for the aqueous extracts of all the cyanobacteria strains studied (Figure 4). Although the MALDI-TOF MS analysis did not reveal the presence of known peptides, our results indicate the production of toxic compounds with diverse chemical natures, since toxic results were obtained with the dichloromethane, the methanolic and the aqueous extracts. Taken into account that the toxic effects on *A. salina* were more evident in the aqueous extract, the risk due to the occurrence of toxic cyanobacteria is high, due to the potential availability of water soluble toxins in the water column.

For toxins such as MC, NOD and CYN, the molecular machinery involved in their production is already elucidated and the presence of toxin-coding genes has been considered an important tool for the study of the potential toxicity of cyanobacteria [24]. All these toxins are produced by a wide range of cyanobacteria genera that have been involved in severe poisoning episodes all over the world [4]. For the strains LEGE 06010 and LEGE 06018, *Leptolyngbya* and *Oscillatoria*, respectively, we could amplify a fragment which showed 99% similarity with the *mcyE* gene of a *Microcystis* sp. strain (Table 2). However, no other genes that constitute the microcystin synthetase gene cluster were identified. The lack of all components of the gene cluster responsible for the production of MC can justify the fact that the toxins were not identified by MALDI-TOF MS since, as it was previously demonstrated that the synthesis of MC requires the presence of the entire *mcy* gene cluster [32]. Rantala *et al.* [33] suggested an ancient origin of MC and reported the loss of genes and, as a consequence, a loss in the ability of some strains to produce toxin. It is known that the transfer of fragments of DNA is common among prokaryotes [34] and, in the particular case of cyanobacteria, genes responsible for toxin production might be subjected to horizontal gene transfer, gene losses [35], transposition, mutagenesis, deletion and recombination [36]. In this case we can hypothesize that deletion events may have occurred resulting in the loss of genes. Besides a possible loss of the genes during evolution, we cannot exclude the possible occurrence of mutations during cultivation of strains. As described by Kaebernick *et al.* [37], mutations within the *mcy* gene cluster might occur during cultivation leading to a decrease in toxicity. Without involving molecular analysis, it was already reported a decrease in the toxicity of strains when maintained in culture. Cyanobacteria seem to be more toxic in the natural environment than under laboratory conditions [38].

Among the *mcy* genes, a region of the *mcyE* has been used as a reliable molecular marker for the detection of MC producers [39]. This gene is related to enzymes that are involved in the synthesis of Adda and addiction of D-glutamate, which are both essential amino acids for the toxicity of MC. In phylogenetic studies it was shown that *mcyE* sequences from different producer genera form their own clusters and remain excluded from horizontal gene transfer [39]. This fact may explain the prevalence of this gene in the strains and the loss of others. Nevertheless, we should point out that we got in our two positive *mcyE* gene isolates, 99% similarity with the *mcyE* of the microcystin synthetase gene cluster from a *Microcystis* sp. CYN06 strain that belongs to a different order. This will be further investigated. The lack of positive results for NOD highlight the production of these toxins only by species of the genus *Nodularia* since this toxin seem to be produced only by species of this genus [36].

Although no peptides such as MC, NOD or other partially known peptides were identified by MALDI-TOF MS, we cannot exclude the presence of other toxins, since ecotoxicological assays were positive for many of the strains and extract types. Existing records on cyanotoxins production are mainly related to freshwater and brackish cyanobacteria [4], but there are some reports about their production by cyanobacteria in marine environments. Some authors reported the production of MC by marine strains of *Synechocystis* and *Synechococcus*, and by filamentous forms of *Oscillatoria*, *Geitlerinema*, *Leptolyngbya*, *Phormidium*, *Pseudoanabaena* and *Spirulina* respectively [12,40,41].

The increasing occurrence of cyanobacteria in large densities at maritime shores resulting from eutrophication and global warming poses a serious threat to humans and ecosystems. Clearly there is a need to increase our knowledge about toxin production by marine cyanobacteria strains, in order to prevent possible adverse effects of its occurrence.

## Figures and Tables

**Table 1:** Strain codes, substrates from which strains were isolated, genera identification (highest similarities of the sequence analysis in GenBank) and accession numbers of the cyanobacteria strains included in this study.

Strain code	Substrate	Genus (16S rRNA highest match NCBI)	% similarity	Accession Nº
LEGE 06005	Sea water	*Synechococcus* sp. UH7	92	HM124558
LEGE 06007	Rock	*Phormidium* sp. HBC9	99	HM124560
LEGE 06008	Rock	*Cyanobium* sp. NS01	99	HM124561
LEGE 06009	Rock	*Leptolyngbya* sp. 0BB32S02	96	HM124563
LEGE 06010	Rock	*Leptolyngbya* sp. 0BB32S02	98	HM124564
LEGE 06011	Rock	*Cyanobium* sp. NS01	98	HM124557
LEGE 06015	*Patella* sp.	*Cyanobium* sp. NS01	99	HM124559
LEGE 06018	Rock	*Oscillatoria* sp. CCAP 1459/26	100	HM124562

**Table 2:** PCR primers used for amplification of 16S rRNA gene for cyanobacteria identification and for the amplification of genes related to cyanotoxins production. A—Individual annealing temperature, B—Reference annealing temperature, bp = base pairs.

Primer	Sequence (5′→3′)	A	B	Size (bp)	Amplified gene	Reference
27F	AGAGTTTGATCCTGGCTCAG	52	60	780	16S rRNA	[22]
809R	GCTTCGGCACGGCTCGGGTCGATA	64				[23]

740F	GGCYRWAWCTGACACTSAGGGA	-	50	754	16S rRNA	[22]
1494R	TACGGCTACCTTGTTACGAC	56				

*mcy*A-Cd F	AAAATTAAAAGCCGTATCAAA	51	59	297	Microcystin synthetase	[28]
*mcy*A-Cd R	AAAAGTGTTTTATTAGCGGCTCAT	43				

HEPF	TTTGGGGTTAACTTTTTTGGGCATAGTC	57	52	472	Microcystin/nodularin synthetase	[23]
HEPR	AATTCTTGAGGCTGTAAATCGGGTTT	55				

PKS M4	GAAGCTCTGGAATCCGGTAA	52	55	650	Cylindrospermopsin polyketide synthase	[29]
PKS M5	AATCCTTACGGGATCCGGTGC	56				[29]

M13	GGCAAATTGTGATAGCCACGAGC	57	55	597	Cylindrospermopsin peptide synthetase	[29]
M14	GATGGAACATCGCTCACTGGTG	57				[29]

**Table 3:** Identification of toxin gene from the samples sequenced using the HEP primers for the strains LEGE 06010 and LEGE 06018. The highest similarities to the sequences in GenBank and Accession numbers are shown.

Strain	Genus	*mcyE* gene (highest match NCBI)	% similarity	Accession Nº
LEGE 06010	*Leptolyngbya* sp.	*Microcystis* sp. CYN06 microcystin synthetase E (*mycE*) gene, complete cds	99%	HM124567
LEGE 06018	*Oscillatoria* sp.	*Microcystis* sp. CYN06 microcystin synthetase E (*mycE*) gene, complete cds	99%	HM124566

**Table 4:** 24 and 48 h LC_50_ values (mg/mL), for the tested cyanobacteria strains and extracts (D: dichloromethane extract; M: methanol extract; A: aqueous extract).

Strain		LEGE	LEGE	LEGE	LEGE	LEGE	LEGE	LEGE	LEGE
Extract		6005	6007	6008	6009	60010	6011	6015	6018
**24h**	**D**	-	-	51.4	-	-	-	-	-
	**M**	51	51.3	50.6	50.9	-	49.9	50.8	51.4
	**A**	49.5	51.1	51	51.3	51.5	50.7	51	49.6

**48h**	**D**	-	-	51.2	-	-	-	-	-
	**M**	50.8	50.7	49.9	50	-	48.3	50.7	51.2
	**A**	49.2	50.9	50.8	51.1	51.5	50.2	51	49.6
